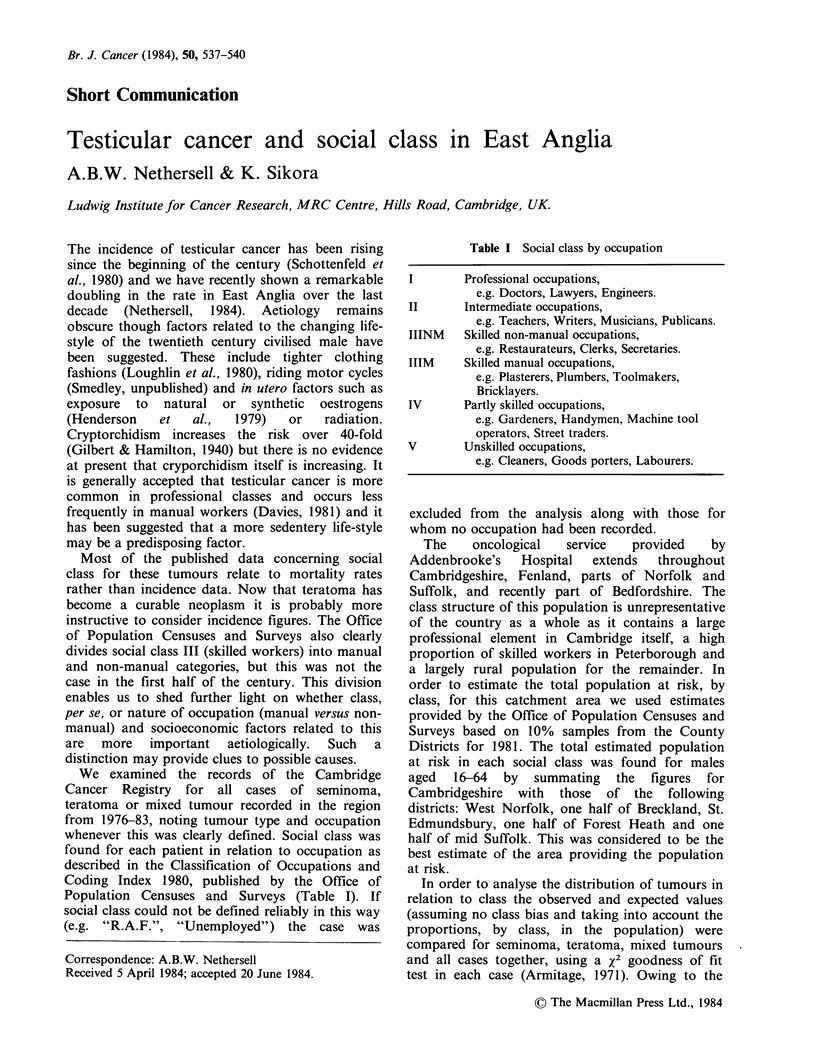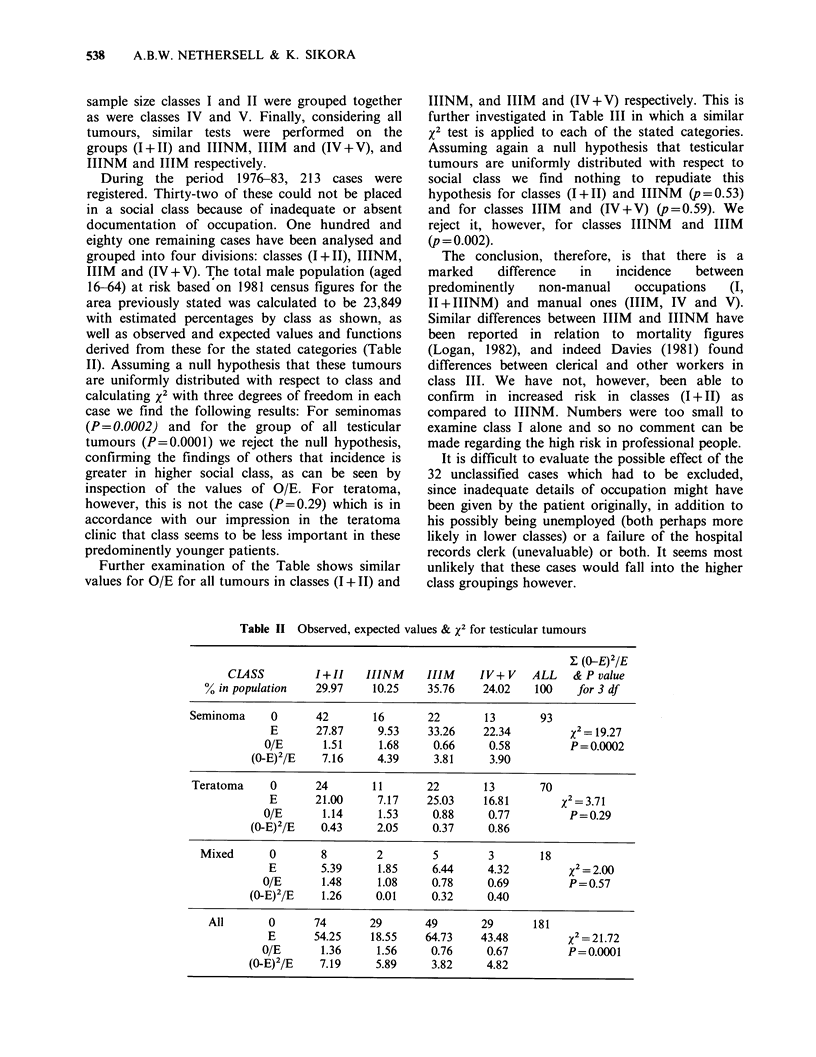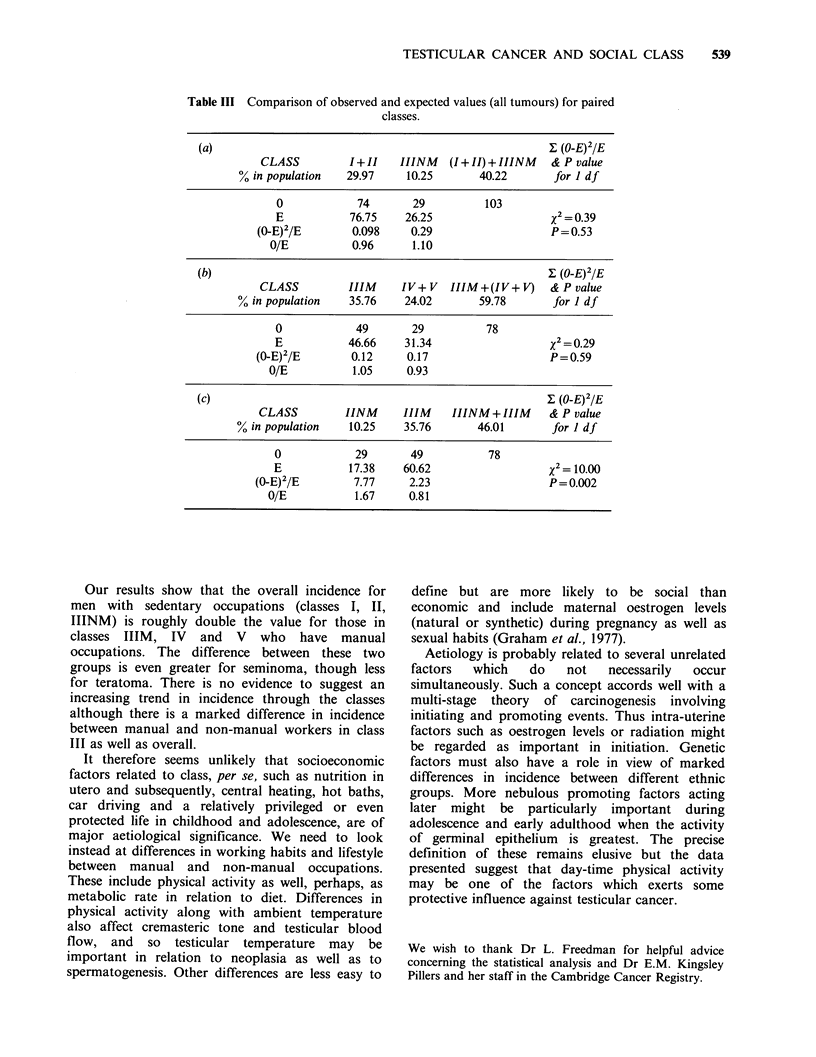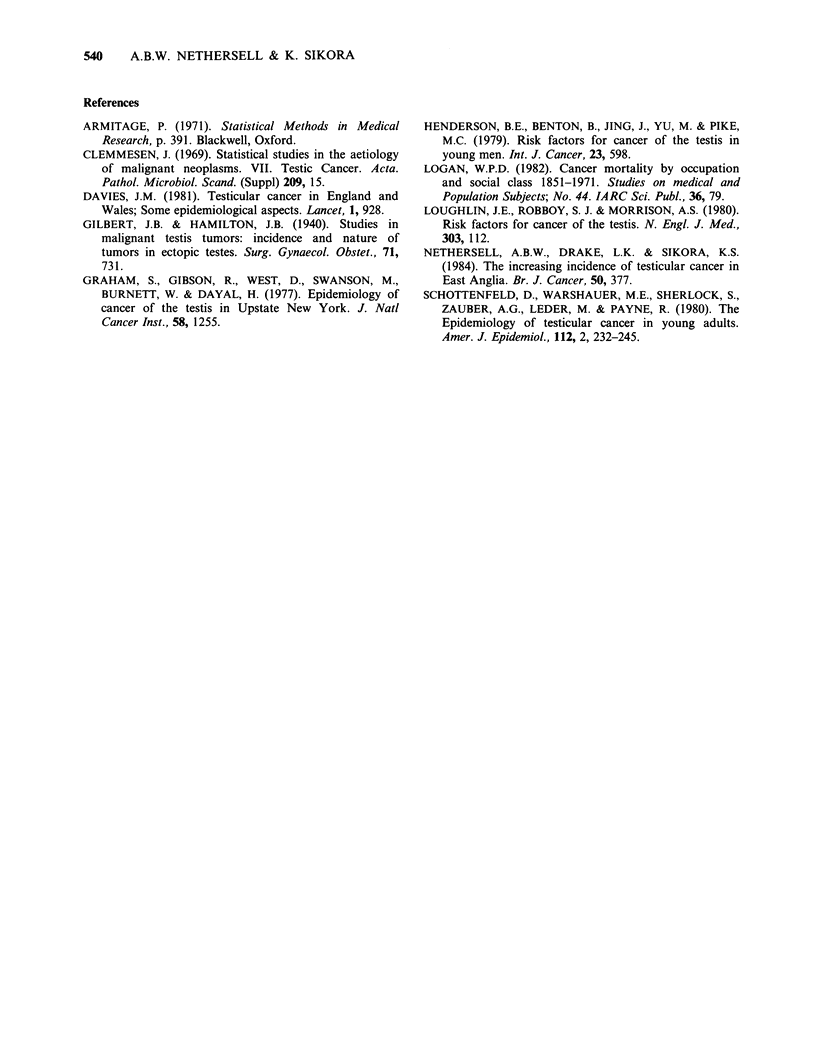# Testicular cancer and social class in East Anglia.

**DOI:** 10.1038/bjc.1984.212

**Published:** 1984-10

**Authors:** A. B. Nethersell, K. Sikora


*Br. J. Cancer* (1984), **50**, 537–540

### Short Communication

### Testicular cancer and social class in East Anglia

A.B.W. Nethersell & K. Sikora

*Ludwig Institute for Cancer Research, MRC Centre, Hills Road, Cambridge, UK.*

The incidence of testicular cancer has been rising since the beginning of the century (Schottenfeld *et al.*, 1980) and we have recently shown a remarkable doubling in the rate in East Anglia over the last decade (Nethersell, 1984). Aetiology remains obscure though factors related to the changing life-style of the twentieth century civilised male have been suggested. These include tighter clothing fashions (Loughlin *et al.*, 1980), riding motor cycles (Smedley, unpublished) and *in utero* factors such as exposure to natural or synthetic oestrogens (Henderson *et al.*, 1979) or radiation. Cryptorchidism increases the risk over 40-fold (Gilbert & Hamilton, 1940) but there is no evidence at present that cryporchidism itself is increasing. It is generally accepted that testicular cancer is more common in professional classes and occurs less frequently in manual workers (Davies, 1981) and it has been suggested that a more sedentery life-style may be a predisposing factor.

Most of the published data concerning social class for these tumours relate to mortality rates rather than incidence data. Now that teratoma has become a curable neoplasm it is probably more instructive to consider incidence figures. The Office of Population Censuses and Surveys also clearly divides social class III (skilled workers) into manual and non-manual categories, but this was not the case in the first half of the century. This division enables us to shed further light on whether class, *per se*, or nature of occupation (manual *versus* non-manual) and socioeconomic factors related to this are more important aetiologically. Such a distinction may provide clues to possible causes.

We examined the records of the Cambridge Cancer Registry for all cases of seminoma, teratoma or mixed tumour recorded in the region from 1976–83, noting tumour type and occupation whenever this was clearly defined. Social class was found for each patient in relation to occupation as described in the Classification of Occupations and Coding Index 1980, published by the Office of Population Censuses and Surveys (Table I). If social class could not be defined reliably in this way (e.g. "R.A.F.", "Unemployed") the case was excluded from the analysis along with those for whom no occupation had been recorded.

**Table I** Social class by occupation

| | |
|---|---|
| I | Professional occupations, e.g. Doctors, Lawyers, Engineers. |
| II | Intermediate occupations, e.g. Teachers, Writers, Musicians, Publicans. |
| IIINM | Skilled non-manual occupations, e.g. Restaurateurs, Clerks, Secretaries. |
| IIIM | Skilled manual occupations, e.g. Plasterers, Plumbers, Toolmakers, Bricklayers. |
| IV | Partly skilled occupations, e.g. Gardeners, Handymen, Machine tool operators, Street traders. |
| V | Unskilled occupations, e.g. Cleaners, Goods porters, Labourers. |

The oncological service provided by Addenbrooke's Hospital extends throughout Cambridgeshire, Fenland, parts of Norfolk and Suffolk, and recently part of Bedfordshire. The class structure of this population is unrepresentative of the country as a whole as it contains a large professional element in Cambridge itself, a high proportion of skilled workers in Peterborough and a largely rural population for the remainder. In order to estimate the total population at risk, by class, for this catchment area we used estimates provided by the Office of Population Censuses and Surveys based on 10% samples from the County Districts for 1981. The total estimated population at risk in each social class was found for males aged 16–64 by summating the figures for Cambridgeshire with those of the following districts: West Norfolk, one half of Breckland, St. Edmundsbury, one half of Forest Heath and one half of mid Suffolk. This was considered to be the best estimate of the area providing the population at risk.

In order to analyse the distribution of tumours in relation to class the observed and expected values (assuming no class bias and taking into account the proportions, by class, in the population) were compared for seminoma, teratoma, mixed tumours and all cases together, using a $\chi^2$ goodness of fit test in each case (Armitage, 1971). Owing to the

Correspondence: A.B.W. Nethersell
Received 5 April 1984; accepted 20 June 1984.

© The Macmillan Press Ltd., 1984

sample size classes I and II were grouped together as were classes IV and V. Finally, considering all tumours, similar tests were performed on the groups (I+II) and IIINM, IIIM and (IV+V), and IIINM and IIIM respectively.

During the period 1976–83, 213 cases were registered. Thirty-two of these could not be placed in a social class because of inadequate or absent documentation of occupation. One hundred and eighty one remaining cases have been analysed and grouped into four divisions: classes (I+II), IIINM, IIIM and (IV+V). The total male population (aged 16–64) at risk based on 1981 census figures for the area previously stated was calculated to be 23,849 with estimated percentages by class as shown, as well as observed and expected values and functions derived from these for the stated categories (Table II). Assuming a null hypothesis that these tumours are uniformly distributed with respect to class and calculating $\chi^2$ with three degrees of freedom in each case we find the following results: For seminomas ($P=0.0002$) and for the group of all testicular tumours ($P=0.0001$) we reject the null hypothesis, confirming the findings of others that incidence is greater in higher social class, as can be seen by inspection of the values of O/E. For teratoma, however, this is not the case ($P=0.29$) which is in accordance with our impression in the teratoma clinic that class seems to be less important in these predominently younger patients.

Further examination of the Table shows similar values for O/E for all tumours in classes (I+II) and IIINM, and IIIM and (IV+V) respectively. This is further investigated in Table III in which a similar $\chi^2$ test is applied to each of the stated categories. Assuming again a null hypothesis that testicular tumours are uniformly distributed with respect to social class we find nothing to repudiate this hypothesis for classes (I+II) and IIINM ($p=0.53$) and for classes IIIM and (IV+V) ($p=0.59$). We reject it, however, for classes IIINM and IIIM ($p=0.002$).

The conclusion, therefore, is that there is a marked difference in incidence between predominently non-manual occupations (I, II+IIINM) and manual ones (IIIM, IV and V). Similar differences between IIIM and IIINM have been reported in relation to mortality figures (Logan, 1982), and indeed Davies (1981) found differences between clerical and other workers in class III. We have not, however, been able to confirm in increased risk in classes (I+II) as compared to IIINM. Numbers were too small to examine class I alone and so no comment can be made regarding the high risk in professional people.

It is difficult to evaluate the possible effect of the 32 unclassified cases which had to be excluded, since inadequate details of occupation might have been given by the patient originally, in addition to his possibly being unemployed (both perhaps more likely in lower classes) or a failure of the hospital records clerk (unevaluable) or both. It seems most unlikely that these cases would fall into the higher class groupings however.

**Table II** Observed, expected values & $\chi^2$ for testicular tumours

| *CLASS* | | *I+II* | *IIINM* | *IIIM* | *IV+V* | *ALL* | $\Sigma\ (0-E)^2/E$ *& P value* |
|---|---|---|---|---|---|---|---|
| *% in population* | | 29.97 | 10.25 | 35.76 | 24.02 | 100 | *for 3 df* |
| Seminoma | 0 | 42 | 16 | 22 | 13 | 93 | |
| | E | 27.87 | 9.53 | 33.26 | 22.34 | | $\chi^2=19.27$ |
| | 0/E | 1.51 | 1.68 | 0.66 | 0.58 | | $P=0.0002$ |
| | $(0\text{-E})^2/\text{E}$ | 7.16 | 4.39 | 3.81 | 3.90 | | |
| Teratoma | 0 | 24 | 11 | 22 | 13 | 70 | |
| | E | 21.00 | 7.17 | 25.03 | 16.81 | | $\chi^2=3.71$ |
| | 0/E | 1.14 | 1.53 | 0.88 | 0.77 | | $P=0.29$ |
| | $(0\text{-E})^2/\text{E}$ | 0.43 | 2.05 | 0.37 | 0.86 | | |
| Mixed | 0 | 8 | 2 | 5 | 3 | 18 | |
| | E | 5.39 | 1.85 | 6.44 | 4.32 | | $\chi^2=2.00$ |
| | 0/E | 1.48 | 1.08 | 0.78 | 0.69 | | $P=0.57$ |
| | $(0\text{-E})^2/\text{E}$ | 1.26 | 0.01 | 0.32 | 0.40 | | |
| All | 0 | 74 | 29 | 49 | 29 | 181 | |
| | E | 54.25 | 18.55 | 64.73 | 43.48 | | $\chi^2=21.72$ |
| | 0/E | 1.36 | 1.56 | 0.76 | 0.67 | | $P=0.0001$ |
| | $(0\text{-E})^2/\text{E}$ | 7.19 | 5.89 | 3.82 | 4.82 | | |

**Table III** Comparison of observed and expected values (all tumours) for paired classes.

| | | | | | |
|---|---|---|---|---|---|
| (*a*) | *CLASS* | *I+II* | *IIINM* | *(I+II)+IIINM* | $\Sigma (0\text{-}E)^2/E$ *& P value* |
| | *% in population* | 29.97 | 10.25 | 40.22 | *for 1 df* |
| | 0 | 74 | 29 | 103 | |
| | E | 76.75 | 26.25 | | $\chi^2=0.39$ |
| | $(0\text{-E})^2/\text{E}$ | 0.098 | 0.29 | | $P=0.53$ |
| | 0/E | 0.96 | 1.10 | | |
| (*b*) | *CLASS* | *IIIM* | *IV+V* | *IIIM+(IV+V)* | $\Sigma (0\text{-}E)^2/E$ *& P value* |
| | *% in population* | 35.76 | 24.02 | 59.78 | *for 1 df* |
| | 0 | 49 | 29 | 78 | |
| | E | 46.66 | 31.34 | | $\chi^2=0.29$ |
| | $(0\text{-E})^2/\text{E}$ | 0.12 | 0.17 | | $P=0.59$ |
| | 0/E | 1.05 | 0.93 | | |
| (*c*) | *CLASS* | *IINM* | *IIIM* | *IIINM+IIIM* | $\Sigma (0\text{-}E)^2/E$ *& P value* |
| | *% in population* | 10.25 | 35.76 | 46.01 | *for 1 df* |
| | 0 | 29 | 49 | 78 | |
| | E | 17.38 | 60.62 | | $\chi^2=10.00$ |
| | $(0\text{-E})^2/\text{E}$ | 7.77 | 2.23 | | $P=0.002$ |
| | 0/E | 1.67 | 0.81 | | |

Our results show that the overall incidence for men with sedentary occupations (classes I, II, IIINM) is roughly double the value for those in classes IIIM, IV and V who have manual occupations. The difference between these two groups is even greater for seminoma, though less for teratoma. There is no evidence to suggest an increasing trend in incidence through the classes although there is a marked difference in incidence between manual and non-manual workers in class III as well as overall.

It therefore seems unlikely that socioeconomic factors related to class, *per se,* such as nutrition in utero and subsequently, central heating, hot baths, car driving and a relatively privileged or even protected life in childhood and adolescence, are of major aetiological significance. We need to look instead at differences in working habits and lifestyle between manual and non-manual occupations. These include physical activity as well, perhaps, as metabolic rate in relation to diet. Differences in physical activity along with ambient temperature also affect cremasteric tone and testicular blood flow, and so testicular temperature may be important in relation to neoplasia as well as to spermatogenesis. Other differences are less easy to define but are more likely to be social than economic and include maternal oestrogen levels (natural or synthetic) during pregnancy as well as sexual habits (Graham *et al.,* 1977).

Aetiology is probably related to several unrelated factors which do not necessarily occur simultaneously. Such a concept accords well with a multi-stage theory of carcinogenesis involving initiating and promoting events. Thus intra-uterine factors such as oestrogen levels or radiation might be regarded as important in initiation. Genetic factors must also have a role in view of marked differences in incidence between different ethnic groups. More nebulous promoting factors acting later might be particularly important during adolescence and early adulthood when the activity of germinal epithelium is greatest. The precise definition of these remains elusive but the data presented suggest that day-time physical activity may be one of the factors which exerts some protective influence against testicular cancer.

We wish to thank Dr L. Freedman for helpful advice concerning the statistical analysis and Dr E.M. Kingsley Pillers and her staff in the Cambridge Cancer Registry.

### References

ARMITAGE, P. (1971). *Statistical Methods in Medical Research*, p. 391. Blackwell, Oxford.

CLEMMESEN, J. (1969). Statistical studies in the aetiology of malignant neoplasms. VII. Testic Cancer. *Acta. Pathol. Microbiol. Scand.* (Suppl) **209,** 15.

DAVIES, J.M. (1981). Testicular cancer in England and Wales; Some epidemiological aspects. *Lancet,* **1,** 928.

GILBERT, J.B. & HAMILTON, J.B. (1940). Studies in malignant testis tumors: incidence and nature of tumors in ectopic testes. *Surg. Gynaecol. Obstet.,* **71,** 731.

GRAHAM, S., GIBSON, R., WEST, D., SWANSON, M., BURNETT, W. & DAYAL, H. (1977). Epidemiology of cancer of the testis in Upstate New York. *J. Natl Cancer Inst.,* **58,** 1255.

HENDERSON, B.E., BENTON, B., JING, J., YU, M. & PIKE, M.C. (1979). Risk factors for cancer of the testis in young men. *Int. J. Cancer,* **23,** 598.

LOGAN, W.P.D. (1982). Cancer mortality by occupation and social class 1851–1971. *Studies on medical and Population Subjects; No. 44. IARC Sci. Publ.,* **36,** 79.

LOUGHLIN, J.E., ROBBOY, S. J. & MORRISON, A.S. (1980). Risk factors for cancer of the testis. *N. Engl. J. Med.,* **303,** 112.

NETHERSELL, A.B.W., DRAKE, L.K. & SIKORA, K.S. (1984). The increasing incidence of testicular cancer in East Anglia. *Br. J. Cancer,* **50,** 377.

SCHOTTENFELD, D., WARSHAUER, M.E., SHERLOCK, S., ZAUBER, A.G., LEDER, M. & PAYNE, R. (1980). The Epidemiology of testicular cancer in young adults. *Amer. J. Epidemiol.,* **112,** 2, 232–245.